# Ensemble learning of myocardial displacements for myocardial infarction detection in echocardiography

**DOI:** 10.3389/fcvm.2023.1185172

**Published:** 2023-10-13

**Authors:** Tuan Nguyen, Phi Nguyen, Dai Tran, Hung Pham, Quang Nguyen, Thanh Le, Hanh Van, Bach Do, Phuong Tran, Vinh Le, Thuy Nguyen, Long Tran, Hieu Pham

**Affiliations:** ^1^ VinUni-Illinois Smart Health Center, VinUniversity, Hanoi, Vietnam; ^2^ College of Engineering and Computer Science, VinUniversity, Hanoi, Vietnam; ^3^ Institute for Artificial Intelligence, VNU University of Engineering and Technology, Hanoi, Vietnam; ^4^ Cardiovascular Center, E Hospital, Hanoi, Vietnam; ^5^ Vietnam National Heart Institute, Bach Mai Hospital, Hanoi, Vietnam; ^6^ Faculty of Information Technology, VNU University of Engineering and Technology, Hanoi, Vietnam

**Keywords:** echocardiography, image segmentation, deep learning, machine learning, myocardial infarction, motion estimation, regional wall motion abnormality, diagnostic ability

## Abstract

**Background:**

Early detection and localization of myocardial infarction (MI) can reduce the severity of cardiac damage through timely treatment interventions. In recent years, deep learning techniques have shown promise for detecting MI in echocardiographic images. Existing attempts typically formulate this task as classification and rely on a single segmentation model to estimate myocardial segment displacements. However, there has been no examination of how segmentation accuracy affects MI classification performance or the potential benefits of using ensemble learning approaches. Our study investigates this relationship and introduces a robust method that combines features from multiple segmentation models to improve MI classification performance by leveraging ensemble learning.

**Materials and Methods:**

Our method combines myocardial segment displacement features from multiple segmentation models, which are then input into a typical classifier to estimate the risk of MI. We validated the proposed approach on two datasets: the public HMC-QU dataset (109 echocardiograms) for training and validation, and an E-Hospital dataset (60 echocardiograms) from a local clinical site in Vietnam for independent testing. Model performance was evaluated based on accuracy, sensitivity, and specificity.

**Results:**

The proposed approach demonstrated excellent performance in detecting MI. It achieved an F1 score of 0.942, corresponding to an accuracy of 91.4%, a sensitivity of 94.1%, and a specificity of 88.3%. The results showed that the proposed approach outperformed the state-of-the-art feature-based method, which had a precision of 85.2%, a specificity of 70.1%, a sensitivity of 85.9%, an accuracy of 85.5%, and an accuracy of 80.2% on the HMC-QU dataset. On the external validation set, the proposed model still performed well, with an F1 score of 0.8, an accuracy of 76.7%, a sensitivity of 77.8%, and a specificity of 75.0%.

**Conclusions:**

Our study demonstrated the ability to accurately predict MI in echocardiograms by combining information from several segmentation models. Further research is necessary to determine its potential use in clinical settings as a tool to assist cardiologists and technicians with objective assessments and reduce dependence on operator subjectivity. Our research codes are available on GitHub at https://github.com/vinuni-vishc/mi-detection-echo.

## 1. Introduction

A myocardial infarction (MI), which is also called a heart attack, happens when blood flow to part of the heart is cut off due to a clot (1) and severely damages the heart tissue. Most of the time, this happens because one or more of the coronary arteries, which bring blood to the heart, are blocked. MI is a serious and potentially life-threatening condition that is the leading cause of death worldwide, affecting 32.4 million people each year (2). In the US alone, about 4 million people visit the emergency room each year with heart symptoms (1). According to a study undertaken by the World Health Organization (3), cardiologists use multiple diagnostic indicators such as pathology outcomes, biochemical marker values, electrocardiography (ECG) findings, and other imaging modalities to diagnose patients with MI (1). However, pathology can only detect dead cells in the heart muscle (3). ECG cannot distinguish between MI and myocardial ischemia symptoms (4), and the specificity of biochemical marker values (cardiac enzymes) is quite low (5). Due to these limitations, none of these techniques are adequate for early MI detection. Another cardiac image modality is echocardiography (ECHO), which is a pivotal tool for a safe and real-time functional assessment of the cardiovascular system (6, 7). It is based on ultrasonography, a noninvasive imaging technique that is incredibly valuable for monitoring and diagnosing patients who are exceedingly vulnerable. Moreover, echocardiography is fast, inexpensive, accessible, portable, and carries the lowest risk among imaging techniques (8, 9). Therefore, the most valuable tool for early diagnosis is an imaging technique known as echocardiography, which is applicable for both clinical and research applications.

With the availability of the MI datasets on echocardiography, machine learning (ML) algorithms have been used to detect MI by extracting features from echocardiography (10). Although they showed promising results, previous studies have largely focused on using features from segmentation models, but the assumption that good segmentation equates to strong classification has yet to be fully substantiated. In addition, current methods are still limited by using only features from a single model, which is a common problem in MI classification and can lead to poor performance on unseen data (11, 12). We conducted experiments to determine the relationship between strong segmentation models and precise MI classification. Our results showed that utilizing the predictions from multiple models through ensemble methods can better identify the patterns and features from echocardiography, resulting in more trustworthy and accurate predictions. Therefore, in this work, we propose a new approach to MI classification by incorporating multiple segmentation models and ensemble learning techniques. Our main contributions are summarized as follows:

∙ Our experiment results showed that there is no strong correlation between good segmentation models and accurate MI classifiers. The finding indicates that highly accurate segmentation of the left ventricle (LV) is not a key condition for accurate MI detection.
∙ We proposed an ensemble method to combine multiple features produced by different LV wall segmentation models. The experimental results show that the proposed ensemble method consistently outperformed the state-of-the-art methods based on single models across all metrics on both the public and external test sets. This result suggests that ensemble learning is successful at complementing the features of multiple feature extractors in a way that a single feature model could not.
∙ The effectiveness of our method was tested on an external dataset obtained from a local clinical source. The results revealed a decline in the model’s performance compared to public data test sets. Possible reasons for the decrease in performance are presented, along with an examination of the implications of these results. Recommendations are also given for enhancing the model’s performance in a clinical environment.
∙ The proposed method showed a higher agreement score (Cohen’s kappa value) than single-feature methods, regardless of whether they used different sets of features. This high level of agreement is an important advantage of the proposed approach as it suggests that the model predictions are subject to variation due to different feature extractors. The high Cohen’s kappa value indicates that our proposed method is reliable and well-suited for use in the classification of MI.

## 2. Related work

MI detection has been a focus of research in the field of medical imaging, with various techniques being proposed to detect abnormalities in LV wall motion (10, 12, 13, 14). With the aim to reduce the cost and time of diagnosis, computer-assisted diagnostic techniques have been developed in recent decades that aim to automate the detection of MI by using image processing and ML techniques (15, 16). Very first studies used active contour-based models, such as (17–19) the snake technique introduced by Kass (20), which uses an elastic curve to detect lines, boundaries, and edges in an image. However, these methods can be impractical or even impossible to use in cases where the LV wall is not visible due to low contrast or a portion of the wall is missing (21). Other MI detection techniques include motion estimation methods (22), which track the displacement of the LV wall, but the accuracy of these methods can depend on the performance of speckle tracking and can lead to unreliable results (23).

Due to limitations in extracting features while using solely image processing techniques, recent studies have shifted to deep learning to extract hidden features from echocardiography images and detect MI. Neural networks such as U-Net (24) and U-Net++ (25) have been widely used for semantic segmentation. Zhang et al. (26), Leclerc et al. (27), Lin et al. (28) presented a large dataset of 2D echocardiography images and proposed a U-Net-based model for accurate segmentation of the LV wall. Degerli et al. (11) utilized the accurate segmentation of the LV wall to detect MI. While studies have shown that deep learning models can be used to detect MI, to the best of our knowledge, no work has explored the direct correlation between a strong segmentation of the LV wall and MI detection.

In addition to developing individual deep learning models, ensemble techniques, which combine the predictions of multiple models, have been explored as a way to improve performance and robustness in the analysis of cardiac functions. For example, Narula et al. (29) improved the performance of the heart’s morphological and functional assessments by using the majority vote from three ML models: support vector machine, random forest, and deep learning. Zhang et al. (30) also increased the diagnostic performance of coronary heart disease screening by stacking ML models. While these studies have shown that ensemble techniques can improve the performance of medical problems, there is still no work that has explored the use of ensemble techniques for MI detection in echocardiography images.

Another major concern while using deep learning models for MI detection is the inconsistency of the results (31). The performance of deep learning models is highly dependent on the quality of the training data, which can be difficult to obtain (32). In addition, the performance of deep learning models can be affected by the choice of the model’s architecture and hyperparameters, which can be difficult to determine (33). To address these issues, we propose a strategy for combining features from different models in order to improve the diagnostic performance of MI detection. The proposed approach is based on the idea that by combining the strengths of various techniques, we can overcome their individual limitations and achieve more accurate and reliable results. We believe that this approach has the potential to significantly advance the field of MI detection and improve patient care.

The remainder of this paper is organized as follows. Section 3 introduces the benchmark HMC-QU dataset, our in-house dataset from E-Hospital, and the framework for determining LV wall motion for MI identification. We will also describe in this section the experimental setting used in this study. Next, Section 4 presents the quantitative and qualitative evaluations of the proposed approach on the HMC-QU dataset and the E-Hospital dataset. We analyze the MI detection performance using various segmentation architectures and classifiers. Finally, Section 5 discusses the experimental results and concludes the paper with some potential research directions.

## 3. Materials and methods

**Figure 1:**
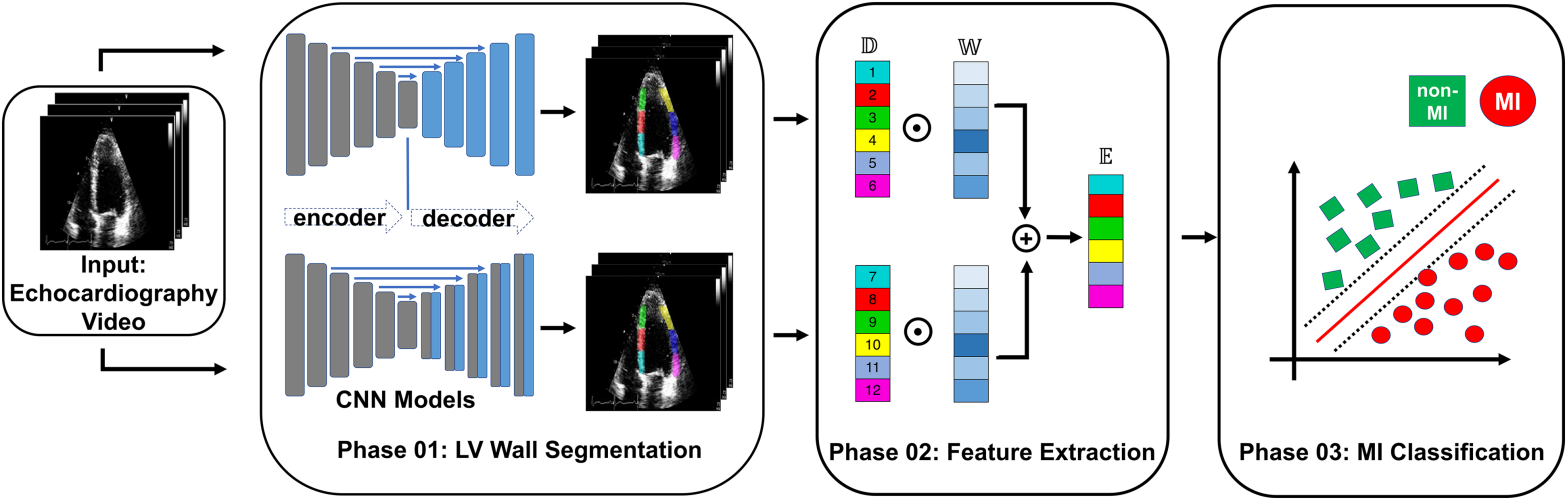
Overview of the proposed MI detection framework. In the phase 01 block, blue blocks represent convolutional layers, gray blocks represent transposed convolutional layers, and blue arrows represent the skip connections between the encoder and decoder. In the phase 02 block, D refers to the displacement of the heart muscle during a cardiac event, W refers to the weight assigned to different features within the ensemble model used for detection, and E refers to the ensemble of features used to identify MI.

In this section, we introduce the proposed ensemble learning framework that addresses the challenge of MI detection. Figure 1 illustrates the overall architecture, consisting of three main phases: LV wall segmentation, feature engineering based on myocardial segmentation displacement, and MI classification using traditional ML classification methods through ensemble models with weighted features from different segmentation models. Below we explain each phase in detail.

∙

Phase 1 - LV wall segmentation: The goal of the first phase is to identify the myocardial boundary, which is a key indicator of heart function. In contrast to Degerli et al. (11), who employed a single segmentation model solely for contouring LV, our approach involves ensembling features extracted from multiple segmentation models. These models excel at segmenting specific regions of the LV wall, providing enhanced accuracy and robustness in our analysis.

∙

Phase 2 - Feature engineering from myocardial segmentation displacement: In this phase, we extract features from the segmented myocardial regions based on displacement over time. These features include measures such as strain, strain rate, and torsion, which are important indicators of myocardial function and can provide insight into the presence of MI. Because we used multiple segmentation models in Phase 1, we proposed a method to combine features from multiple segmentation results.

∙

Phase 3 - MI classification: In the final phase, traditional classification ML methods such as support vector machines, random forests, and logistic regression, are explored to detect MI from the extracted features. To further improve the performance of the proposed model, we also implement an ensemble learning approach by weighting the features from the different segmentation models. The weighting step takes into account the segmentation accuracy of each segmentation model. The performance of the proposed framework will be evaluated using common metrics, including sensitivity, specificity, precision, F1 score, and accuracy.

### 3.1. DATASET

**Figure 2:**
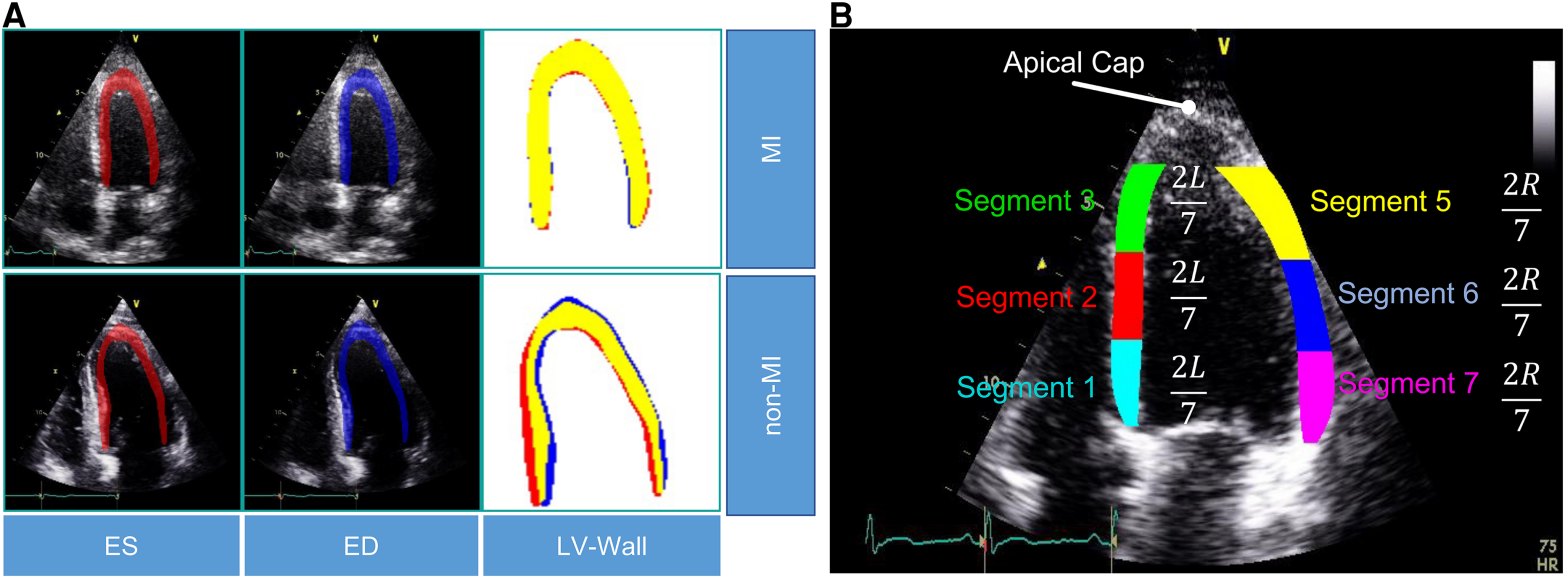
(**A**) Segmentation mask of the LV wall for both an end-systolic frame and an end-diastolic frame, with one case representing an MI and the other case representing a non-MI case in the HCM-QU dataset. (**B**) Six segments of the LV wall may be used to detect signs of an MI. The label “L” represents the length from the bottom left corner to the apex of the LV, and the label “R” represents the length from the bottom right corner to the apex of the LV.

In this study, we used the public HMC-QU dataset (11) as the training and validation sets. The HMC-QU dataset, consisting of 2D echocardiography recordings for the detection of MI, was established through collaboration between cardiologists from the Hamad Medical Corporation, researchers from Qatar University, and Tampere University. The ultrasound machines used to acquire the data were made by GE Healthcare, and the recordings have spatial and temporal resolutions that vary from 422×636 to 768×1024 and 25 frames per second, respectively. The collection includes 162 Apical Four Chambers (A4C) ultrasound videos acquired between 2018 and 2019, but for the purposes of this study, a subset of 109 ultrasound videos was used, resulting in a total of 2,349 images from 72 MI patients and 37 non-MI subjects. The remaining 53 ultrasound videos were excluded because they did not include the entire LV wall, which was necessary for cardiologists to evaluate. As depicted in Figure 2A, the non-MI and MI cases have two frames: end-systolic and end-diastolic. The MI case has a larger overlap region compared to the non-MI sample, which is an indicator of MI, though the determination of MI requires the evaluation of multiple frames.

To evaluate the effectiveness of our proposed method, we collected a dataset of patient records from E-Hospital, a local clinical site in Vietnam. The institutional review board at the clinical site approved the study, and we followed ethical guidelines in collecting and processing the data. Since this retrospective study did not have any influence on clinical care or workflow at the clinical site, and all patient-identifiable information has been thoroughly eliminated from the data, the requirement to obtain informed patient consent was waived. We obtained 200 patient records from E-Hospital for this study, without specific selection criteria, covering the period from 2020 to 2021. The data were acquired using the Philip Affiniti 70G ultrasound imaging system, and we carefully examined all the data to ensure high-quality and visible anatomy structures. Out of 200 samples, cardiologists have removed samples that did not meet quality standards (e.g. correct orientation, no artifacts, and complete cardiac cycle), resulting in a total of 60 data samples being retained for further analysis. Among these samples, 36 corresponded to patients with MI, while the remaining 24 samples represented non-MI patients. To ensure the accuracy and consistency of MI region annotations, three experienced doctors were involved in the annotation process. Additionally, a separate group of three doctors verified the annotations for precision and uniformity, the final decision is determined by the decision of the most experienced doctors. The annotated data, stored in the Digital Imaging and Communications in Medicine (DICOM) format, was utilized for testing our proposed model. It is important to note that the dataset exclusively comprises MI annotations and was employed as an independent test set to evaluate the performance of our model. Detailed statistical information for both the HMC-QU and E-Hospital datasets can be found in Table 1.

**Table 1:** Sample counts of MI and non-MI patients by LV wall segments in HMC-QU and E-Hospital datasets.

LV wall segments	HMC-QU dataset		E-Hospital dataset	
	# MI patients	# non-MI patients	# MI patients	# non-MI patients
Segment-1	24	85	14	46
Segment-2	43	66	15	45
Segment-3	59	50	12	48
Segment-5	44	65	7	53
Segment-6	25	84	13	47
Segment-7	15	94	15	45
Patient-based	72	37	36	24

### 3.2. Feature engineering

Similar to Degerli et al. (11), we extract the motion feature by performing two consecutive steps: LV segmentation and feature calculation. The classification of MI is performed by utilizing features extracted from the complete set of cardiac cycle images. Below we explain these two steps in detail.

*LV segmentation.* We use a U-Net-like convolutional network that consists of an encoding and down-sampling path, followed by a decoding and up-sampling path with skip links (24). The model’s output is a mask of the LV obtained through the Softmax function. During the training phase, we use cross-entropy as the loss function to optimize the model’s parameters. Cross-entropy, which is defined as a measure of the difference between two probability distributions for a given random variable or set of events, is widely used for classification objectives. The binary cross-entropy is defined as

(1) $$L_{BCE}(y,\hat{y})=-(y\log(\hat{y})+(1-y)\log(1-\hat{y})),$$

where $\hat{y}$ is the predicted value by the prediction model.

We analyze the segments of the LV wall to detect possible MI signatures. A standardized model (6) recommends dividing the LV wall into seven segments, as depicted in Figure 2B. However, in our analysis, we only consider six of them, as the apical cap does not exhibit inward motion activity and should be skipped for the A4C view.

*Feature calculation.* For MI detection, we extract the displacement of the endocardial boundary points from the six segments (Figure 2B). By evaluating the rate of displacement from the captured global motion of the LV wall, we aim to mimic the typical diagnosis of cardiologists, who assess segments that show a lack of motion as abnormal.

After segmenting the LV wall, we further extract its inner border to define the endocardial boundary, which is then divided into six segments. In order to capture the boundary segment motion more precisely, we estimate the motion between two frames at two different time points, t, and $t_{r}$ in the video, in our case, $t_{r}$ is the first frame of a video sequence and t is arbitrary time point. In frame at time t, we select a segment indexed by s and take N sampled pixels $p\in \{(x_{1},y_{1}),(x_{2},y_{2}),\ldots ,(x_{N},y_{N})\}$ in this segment. These pixels are obtained by dividing each segment into N−1 equally spaced intervals and selecting N pixels accordingly. This ensures that the distance between two consecutive pixels remains consistent within each segment. For each segment s, we calculate the pair-wise distances $d_{s,t}$ between two frame time $t_{r}$ and t, and the segment displacement for each segment s at time t is then calculated using L1 norm as follows

(2) $$d_{s,t}=\frac{1}{N}\sum_{n=1}^{N}|x_{n}^{s,t_{r}}-x_{n}^{s,t}|+|y_{n}^{s,t_{r}}-y_{n}^{s,t}|.$$

For a video containing T frames, we obtain T−1 displacement values for each segment s. Finally, we take the maximum pixel displacement of each segment, from the displacement curves, and normalize it to unity. The motion feature, $MF\in R^{6}$, is then calculated by taking the maximum displacement value over time as

(3) $$MF=\max_{t}(\{d_{s,t}|t\in \{1,2,\ldots ,T-1\},t\neq t_{r},s\in \{1,2,\ldots ,6\}\}.$$

### 3.3. Ensemble of features

Ensemble learning, which involves training a group of different classifiers and combining their predictions, has been shown to improve model performance and robustness (34). During the process of feature engineering, we discovered that different segmentation models perform better on different segments (as shown in Table 2). This meant that the performance of the classifier was limited by the performance of the segmentation model. We chose to combine the displacement vectors instead of using complete segmentation maps because the displacement feature vectors have a lower dimensionality, which reduces the saturation effect of weighting. To enable the classifier to benefit from multiple features, we calculated the accumulated vector by summing multiple feature vectors based on the accuracy of the segmentation models on the validation set. This approach has several benefits: 1) the weighting method does not require any training, so it can be used universally with different classifiers; 2) conventionally, stacking multiple feature vectors can increase the dimensionality of the accumulated feature vector, making it harder to train the classifier and limiting the number of features that can be used. In contrast, the weighted sum vector has a fixed dimension regardless of the number of segmentation models used.

**Table 2:** Performance in IoU of different segmentation models on different segments of the LV wall, IoU for whole LV wall, mean Hausdorff distance between consecutive frames of whole LV wall, and Mean Absolute Distance of whole LV wall on HMC-QU validation set.

Models	Segment						Full LV		
	1	2	3	5	6	7	IoU	Hausdorff	MAD
Unet++	$0.851_{\pm .08}$	$0.905_{\pm .05}$	$0.873_{\pm .04}$	$0.823_{\pm .09}$	$0.873_{\pm .06}$	$0.932_{\pm .07}$	$0.871_{\pm .05}$	$4.08_{\pm 1.89}$	$1.32_{\pm 0.71}$
Unet	$0.868_{\pm .05}$	$0.845_{\pm .06}$	$0.877_{\pm .02}$	$0.905_{\pm .03}$	$0.979_{\pm .08}$	$0.894_{\pm .04}$	$0.876_{\pm .02}$	$3.75_{\pm 2.12}$	$1.22_{\pm 1.34}$
PAN	$0.858_{\pm .07}$	$0.938_{\pm .09}$	$0.729_{\pm .03}$	$0.901_{\pm .07}$	$0.864_{\pm .06}$	$0.845_{\pm .04}$	$0.853_{\pm .07}$	$4.21_{\pm 2.73}$	$1.11_{\pm 0.52}$
LinkNet	$0.895_{\pm .06}$	$0.893_{\pm .02}$	$0.880_{\pm .03}$	$0.868_{\pm .05}$	$0.809_{\pm .07}$	$0.859_{\pm .02}$	$0.867_{\pm .04}$	$4.65_{\pm 3.21}$	$1.54_{\pm 1.89}$
DeepLabV3	$0.870_{\pm .03}$	$0.766_{\pm .09}$	$0.854_{\pm .06}$	$0.883_{\pm .04}$	$0.939_{\pm .05}$	$0.839_{\pm .07}$	$0.860_{\pm .06}$	$4.33_{\pm 1.45}$	$1.02_{\pm 1.07}$

Bold indicates the best performance.

Formally, given n motion features vector $MF\in R^{6}$ extracted from n segmentation model with an objective metric for different segments $M\in R^{6}$. The weighted coefficient W is calculated as

(4) $$W_{i}=\frac{M_{i}}{\sum_{i=i}^{n}M_{i}}.$$

Accumulated feature vector $MF_{\mathrm{acc}}\in R^{6}$ is calculated as

(5) $$MF_{\mathrm{acc}}=\sum_{i=i}^{n}MF_{i}⊙W_{i}.$$

### 3.4. MI detection

In the final step of the pipeline, we use ML to detect MI in an echocardiogram. To do this, we employ four conventional supervised ML techniques, including support vector machine (35), logistic regression (36), decision trees (37), and k-nearest neighbor (38). These techniques were chosen over more complex deep learning methods due to the small and imbalanced nature of our dataset, as well as the fact that the extracted features are more suited to simpler analysis. To fairly evaluate the performance of these classifiers, we use a stratified 5-fold cross-validation scheme. The details of their configuration, training, and testing are discussed in the following section.

### 3.5. Evaluation metrics

*LV segmentation.* The Intersection over Union (IoU) metric, also known as the Jaccard index, is used to measure the overlap between the target mask and the prediction output. This metric is similar to the Dice coefficient, which is frequently used as a loss function during training. The IoU is calculated by dividing the intersection of the target and prediction by their union. It is written as

(6) $$\mathrm{IoU}=\frac{\text{target}\cap \text{\textbackslash{},prediction}}{\text{target}\cup \text{\textbackslash{},prediction}}.$$

*MI detection.* For the MI detection, we classify infarcted subjects as class-positive (MI) and normal, non-MI subjects as class-negative. In this case, the confusion matrix is formed as follows: TN is the number of correctly predicted non-MI subjects, TP is the number of correctly predicted MI patients, FN is the number of incorrectly detected MI patients as non-MI subjects, and FP is the number of incorrectly detected non-MI subjects as MI patients. The elements of the confusion matrix are calculated per video for MI detection. The standard performance evaluation metrics are defined as

(7) $$\mathrm{Sensitivity}=\frac{\mathrm{TP}}{\mathrm{TP}+\mathrm{FN}},$$

(8) $$\mathrm{Specificity}=\frac{\mathrm{TN}}{\mathrm{TN}+\mathrm{FP}},$$

(9) $$\mathrm{Accuracy}=\frac{\mathrm{TP}+\mathrm{TN}}{\mathrm{TP}+\mathrm{TN}+\mathrm{FP}+\mathrm{FN}},$$

(10) $$\mathrm{Precision}=\frac{\mathrm{TP}}{\mathrm{TP}+\mathrm{FP}},$$

(11) $$F(\beta )=(1+\beta^{2})\frac{\text{Precision}\times \text{Sensitivity}}{\beta^{2}\times \text{Precision}+\text{Sensitivity}},$$

where TP, FP, TN, and FN denote the numbers of true positive, false positive, true negative, and false negative cases, respectively. Sensitivity (also known as recall) is the ratio of correctly detected MI patients to all MI patients. Specificity is the ratio of correctly classified non-MI subjects to all non-MI subjects. Precision refers to the number of correctly detected MI patients over the total number of correctly detected samples. Accuracy is the ratio of correctly detected samples. F1 score is calculated as the harmonic average of precision and sensitivity, with a weighting of β=1 in the dataset.

*Prediction reliability.* In addition to the performance of the prediction model, the reliability of the model is a crucial criterion for ML models used in medical applications. In this case, reliability can be interpreted as the consistency of the classifier’s output regardless of the use of different feature extractors or combinations of feature extractors. We quantify this criterion by calculating the Cohen’s Kappa (39) coefficient of the model’s output based on the feature extractor. In this work, Cohen’s Kappa coefficient is calculated for three scenarios:

∙ Agreement score between different predictors that use a single feature extractor.
∙ Agreement score between ensemble model using a different set of feature extractors.
∙ Agreement score between cardiologist experts on single echo.

### 3.6. Experimental settings

*LV wall segemtation.* In our scientific paper, we evaluated the model’s performance on the HMC-QU dataset, consisting of 119 ultrasound videos. To ensure a robust evaluation, we employed a stratified 5-fold cross-validation scheme. Specifically, we divided the dataset into five folds while maintaining the distribution of ultrasound videos across different classes. During each fold, we trained the model using 80% of the ultrasound videos (95 ultrasound videos) and evaluated its performance on the remaining 20% holdout ultrasound videos (24 ultrasound videos) that were unseen during training.

During the training process, we observed that models converged after 30 epochs. In order to provide an extended training procedure and further explore the potential improvements, we decided to add an additional 20 epochs resulting total of 50 epochs for each model. The learning rate of models is $1\times 10^{-4}$. The model was implemented in Pytorch and optimized using the Adam optimizer with parameters $\beta_{1}=0.9$ and $\beta_{2}=0.999$. The training and testing were conducted on a GTX 3090 GPU, and the number of total parameters ranged from 1 million to 23 million.

In our experiments, we explored five architectures, including UNet (24), UNet++ (25), LinkNet (40), DeepLabV3 (41), and PAN (42), as well as five encoder architectures, including resnet18 (43), vgg11 (44), densenet121 (45), efficientnet-b0 (46), and inceptionv4 (47). The segmentation performance was evaluated on a pixel level using the IoU score metric described in Section 3.5.

*MI detection.* In this experiment, we applied four supervised ML techniques for binary classification, including support vector machine (SVM), logistic regression (LR), decision trees (DT), and k-nearest neighbor (KNN). For training and selecting optimal hyperparameters for each model, we utilized the HMC-QU dataset. To divide the dataset into training and validation sets, we employed a 5-fold cross-validation scheme. Subsequently, the best-performing model was tested on the E-Hospital data as an external test set. It’s important to note that while there may be a domain shift issue when employing the same weights for segmentation models in both the HMC-QU data and the E-Hospital data, collecting segmentation ground truth for the E-Hospital dataset would be impractical due to its time-consuming nature.

To determine the optimal hyperparameters for each classifier, we do a grid search on the following parameter options. The first classifier is SVM with options including the regularization parameter C (4 options ranging from 0.01 to 100), and probability estimation enabled. The second classifier used Logistic Regression with options such as the regularization parameter C (ranging from 0.01 to 100), penalty type (L1 or L2), solver method (“liblinear”), and maximum iteration limit. For the third model, the DT was employed with options including maximum depth (5, 10, 25, None), minimum samples required for splitting (2, 5, 10), and default random state. The last model was KNN with the number of neighbors (2, 5, 10, 25, 50) as the sole hyperparameter. Through careful evaluation and comparison of these models with their respective parameter options, we were able to identify the most suitable hyperparameters for our specific problem. We then used the trained model to predict the labels for the instances in the test set. The performance of each model was evaluated using a variety of metrics, such as accuracy, precision, and recall. Finally, we compared the performance of the different models to determine which one achieved the best results. In order to further assess the significance of segmentation in MI detection, we also conducted a benchmark analysis using different classifiers. The evaluation involved comparing the results obtained from ground truth segmentation maps with those obtained from ensembles of ground truth and other segmentation models.

*Model reliability analysis.* To evaluate the reliability of the predictions made by the different classification methods, we first randomly selected 20 examples from the test set. We then calculated the agreement score within each classification method (SVM, LR, DT, and KNN) that used only features derived from a single segmentation model (eg. an agreement between combinations of SVM with segmentation models such as UNet, PAN, …). Each classification method was trained using four different sets of features. For the ensemble methods, we also calculated the agreement score between the four models, each of which was created using a different pair of features (e.g. agreement between combinations of SVM with segmentation models such as UNet and PAN, UNet and Deeplabv3, …). Finally, we computed the human expert agreement score using the labels provided by three experts on the same echo samples. This allowed us to measure the similarity of the output of the feature extractors and classifiers on the same input.

## 4. Results

In this section, we present the results of our experiments on LV wall segmentation and MI classification using our proposed method on the HMC-QU validation test and a separate E-Hospital test set, respectively. We also evaluate the performance of our method through various metrics and techniques. First, we report the performance of our model on LV wall segmentation on the HMC-QU validation test on different LV wall segments, where we compare the state-of-the-art methods in the field. Next, we present the MI classification results on the HMC-QU and E-Hospital datasets and compare our model’s performance against the baseline and other existing approaches. Finally, we discuss the reliability of our model by analyzing its agreement scores compared to existing methods and human experts.

### 4.1. LV wall segmentation on HMC-QU validation test

Table 2 shows the LV wall segmentation averages (mean) results for 5-fold CV with different network architectures and encoders. The results indicate a stable IoU score between various types of models. The maximum IoU score can get is **0.876%** when using UNet architecture and resnet18 encoder. Despite the fact that there is no significant variation in segmentation performance during validation, the segmentation models have different scores on different heart segments, as we have discussed in Section 3.3.

### 4.2. MI classification on test set

Table 3 demonstrates that the performance of concatenated features from multiple segmentation models is superior compared to a single segmentation model and the performance across several classifications is consistent. Overall, the model achieved better performance by using ensemble features.

∙ Models with the same classifier performed similarly when using only one feature, on the other hand, ensemble models show better performance. Using SVM models, for instance, PAN or UNet segmentation features yielded comparable F1 scores of 0.829 for PAN and 0.831 for UNet. In contrast, the ensemble SVM model performed better with F1 score of 0.925. This improvement is rather uniform across classifiers by a substantial margin.
∙ Upon comparing the performance of several classifiers, we found that there exist discrepancies in the approaches employed. Using an ensemble of two or three features, LR provided the best performance with F1 score of 0.942, while SVM, DT, and KNN only achieved F1 scores of 0.925, 0.910, and 0.894, respectively. When more features are added to the ensemble, however, this result becomes inconsistent, with LR’s F1 score dropping to 0.905 and DT’s F1 score reaching 0.931. This finding suggests that classifier selection should be based on experimentation with all classifiers.
∙ Regarding the number of features used to construct the model ensemble. We have experimented with combining two and three features. We discovered that combining additional features does not produce better results. In reality, adding more features to a model might diminish its performance. In the case of the SVM classifier, adding three features decreased the F1 score from 0.925 to 0.903. This could indicate that adding more features to an ensemble could eventually increase the noise to the final features, eradicating the effectiveness of the advantage segment.
∙ Our ensemble method showed significantly better performance compared to the average ensemble approach. our method achieved an F1 score of 0.925, while the average ensemble approach only achieved an F1 score of 0.806. This demonstrates the effectiveness of choosing the right regions from the LV wall, rather than average regions altogether.

**Table 3:** MI accuracy based on segmentation features with different classifiers.

Classifier	Model	IoU	Sensitivity	Specificity	Precision	F1 score	ACC
SVM	Single-PAN	$0.853_{\pm .03}$	$0.829_{\pm .07}$	$0.633_{\pm .11}$	$0.867_{\pm .11}$	$0.829_{\pm .06}$	$0.753_{\pm .05}$
SVM	Single-Unet	$0.871_{\pm .02}$	$0.764_{\pm .05}$	$0.857_{\pm .06}$	$0.914_{\pm .06}$	$0.831_{\pm .06}$	$0.797_{\pm .07}$
SVM	AE	-	$0.740_{\pm .06}$	$0.805_{\pm .07}$	$0.891_{\pm .07}$	$0.806_{\pm .07}$	$0.761_{\pm .08}$
SVM	WE	-	$0.947_{\pm .08}$	$0.783_{\pm .08}$	$0.913_{\pm .07}$	$0.925_{\pm .08}$	$0.890_{\pm .09}$
LR	Single-PAN	$0.853_{\pm .03}$	$0.767_{\pm .09}$	$0.783_{\pm .10}$	$0.902_{\pm .08}$	$0.816_{\pm .04}$	$0.756_{\pm .03}$
LR	Single-Unet	$0.871_{\pm .02}$	$0.769_{\pm .08}$	$0.960_{\pm .07}$	$0.967_{\pm .09}$	$0.851_{\pm .03}$	$0.820_{\pm .05}$
LR	AE	-	$0.771_{\pm .07}$	$0.776_{\pm .04}$	$0.858_{\pm .08}$	$0.806_{\pm .10}$	$0.774_{\pm .11}$
LR	WE	-	$0.941_{\pm .11}$	$0.883_{\pm .07}$	$0.950_{\pm .10}$	$0.942_{\pm .06}$	$0.914_{\pm .10}$
DT	Single-PAN	$0.853_{\pm .03}$	$0.787_{\pm .03}$	$0.760_{\pm .11}$	$0.907_{\pm .08}$	$0.836_{\pm .10}$	$0.784_{\pm .07}$
DT	Single-LinkNet	$0.867_{\pm .02}$	$0.773_{\pm .08}$	$0.607_{\pm .08}$	$0.809_{\pm .09}$	$0.788_{\pm .09}$	$0.722_{\pm .09}$
DT	AE	-	$0.726_{\pm .07}$	$0.795_{\pm .03}$	$0.887_{\pm .08}$	$0.794_{\pm .04}$	$0.749_{\pm .04}$
DT	WE	-	$0.926_{\pm .04}$	$0.733_{\pm .10}$	$0.898_{\pm .10}$	$0.910_{\pm .07}$	$0.870_{\pm .10}$
KNN	Single-PAN	$0.853_{\pm .03}$	$0.833_{\pm .08}$	$0.893_{\pm .07}$	$0.942_{\pm .08}$	$0.883_{\pm .03}$	$0.851_{\pm .05}$
KNN	Single-LinkNet	$0.867_{\pm .02}$	$0.812_{\pm .06}$	$0.783_{\pm .09}$	$0.908_{\pm .07}$	$0.844_{\pm .08}$	$0.787_{\pm .08}$
KNN	AE	-	$0.769_{\pm .04}$	$0.827_{\pm .06}$	$0.902_{\pm .06}$	$0.822_{\pm .05}$	$0.794_{\pm .03}$
KNN	WE	-	$0.899_{\pm .03}$	$0.750_{\pm .07}$	$0.910_{\pm .06}$	$0.894_{\pm .09}$	$0.849_{\pm .04}$
SVM	Ground-Truth (GT)	**1.00**	$0.820_{\pm .07}$	$0.844_{\pm .08}$	$0.922_{\pm .08}$	$0.864_{\pm .08}$	$0.827_{\pm .09}$
SVM	GT-Unet-AE	-	$0.806_{\pm .10}$	$0.785_{\pm .06}$	$0.892_{\pm .04}$	$0.844_{\pm .07}$	$0.800_{\pm .04}$
SVM	GT-Unet-WE	-	$0.844_{\pm .08}$	$0.768_{\pm .06}$	$0.891_{\pm .07}$	$0.864_{\pm .07}$	$0.820_{\pm .07}$
SVM	GT-PAN-AE	-	$0.781_{\pm .12}$	$0.801_{\pm .03}$	$0.887_{\pm .03}$	$0.827_{\pm .09}$	$0.789_{\pm .10}$
SVM	GT-PAN-WE	-	$0.849_{\pm .08}$	$0.817_{\pm .05}$	$0.908_{\pm .09}$	$0.875_{\pm .06}$	$0.839_{\pm .07}$

The ensemble row shows the model that utilizes an ensemble of upper features on the HMC-QU dataset. The abbreviations ‘AE' and ‘WE’ denote the averaging ensemble and weighting ensemble, respectively. Bold indicates the best performance.

*Correlation between good segmentation models and accurate MI classifications.* In our experiment, we evaluated the performance of four different MI detection algorithms. Each algorithm used features from a weaker segmentation approach (PAN), and more accurate segmentation techniques (UNet and LinkNet). As shown in the Table 3, While LinkNet and UNet achieved higher IoU scores of 0.871 and 0.867, respectively, algorithms that use PAN features still performed better than LinkNet and UNet with F1 score of 0.836 for DT-PAN and 0.788 DT-LinkNet. When utilizing ground truth as a feature in both single and ensemble classifiers, we observed a consistent phenomenon. Specifically, the MI classification results were lower when employing ground truth segmentation. These results suggest that, while good segmentation is important for MI detection, it is not the only factor that determines the overall performance of the algorithm. In this case, the combination of advanced segmentation and effective feature extraction/classification techniques appears to be crucial for achieving optimal results.

*Comparison with previous state-of-the-art method.* Table 4 shows the performance of our ensemble models and the previous state-of-the-art method from (11). In general, our ensemble LR model with two PAN features outperforms the state-of-the-art model presented in (11) by a significant margin. The F1 score for our model is 0.09 higher than the previous model, indicating that it is able to more accurately predict the outcome of interest. Additionally, when considering other evaluation metrics such as sensitivity, specificity, precision, and accuracy, our model consistently outperforms the model from (11). This suggests that using a larger number of features can be beneficial for improving the performance of an ML model.

**Table 4:** Comparison of performance metrics for our model and state-of-the-art method in a 5-fold cross-validation setting on the HMC-QU dataset, both methods use the same experimental settings.

Classifier	Sensitivity	Specificity	Precision	F1 score	ACC
LDA (11)	0.785	0.701	0.838	0.806	0.756
RF (11)	0.802	0.718	0.859	0.825	0.774
DT (11)	0.790	0.586	0.804	0.794	0.726
DT (**Ours**)	0.926	0.733	0.898	0.910	0.870
SVM (11)	0.859	0.701	0.855	0.852	0.802
SVM (**Ours**)	**0.947**	**0.783**	**0.913**	**0.925**	**0.890**

Bold indicates the best performance.

*Performance of classification models on external test set.* Table 5 shows the result of our ensemble model in comparison with the single-feature model on the local clinical site, E-Hospital. The performance of the ensemble model on the external local clinical test set was found to be highly correlated with the results obtained on the public data test set. In terms of the F1 score, the ensemble model achieved a score of 0.824 on the local clinical test set, which was significantly higher than the score from single-feature and average ensemble models. This demonstrates the consistency of our methods on different datasets. Additionally, the best ensemble model had a sensitivity, of 0.806 on the local clinical test set, which was again higher than the single feature model and an average ensemble model. Overall, these results suggest that the MI classification model is reliable and effective in identifying MI events in both familiar and novel datasets.

**Table 5:** MI accuracy based on segmentation features with different classifiers, ensemble row indicate model using an ensemble of upper features on E-Hospital dataset.

Classifier	Model	Sensitivity	Specificity	Precision	F1 score	ACC
SVM	Single-PAN	0.722	0.583	0.722	0.722	0.667
SVM	Single-Unet	0.694	0.667	0.758	0.725	0.683
SVM	AE	0.667	0.750	0.800	0.727	0.700
SVM	WE	**0.806**	0.625	0.763	**0.784**	0.733
LR	Single-PAN	0.694	0.625	0.735	0.714	0.667
LR	Single-Unet	0.611	**0.792**	0.815	0.698	0.683
LR	AE	0.722	0.708	0.788	0.754	0.717
LR	WE	0.778	0.750	0.824	**0.800**	**0.767**
DT	Single-LinkNet	0.722	0.667	0.765	0.743	0.700
DT	Single-PAN	0.694	0.750	**0.806**	0.746	0.717
DT	AE	0.722	0.708	0.788	0.754	0.717
DT	WE	**0.806**	0.667	0.784	**0.795**	0.750
KNN	Single-LinkNet	0.667	0.542	0.686	0.676	0.617
KNN	Single-PAN	0.722	0.583	0.722	0.722	0.667
KNN	AE	0.667	0.708	0.774	0.716	0.683
KNN	WE	**0.806**	0.625	0.763	**0.784**	0.733

The abbreviations “AE” and “WE” denote the averaging ensemble and weighting ensemble, respectively. The single models used are equivalent to Degerli et al. (11) when using features from different segmentation models. Bold indicates the best performance.

We also discovered that the external test on the local clinical dataset yielded lower scores compared to the public test set. Specifically, the model performed with an F1 score of 0.824 on the local clinical dataset, while it achieved an F1 score of 0.942 on the public test set. This suggests that the model may not generalize as well to the local clinical dataset, possibly due to differences in the distribution of the data or the specific characteristics of the patient population, discrepancies in ultrasound machines, or examination protocols represented in the dataset. Solving this problem may be a task for future work. There are several potential approaches that could be pursued in order to address this issue, some possible approaches could include using different types of ML algorithms, collecting additional data, or fine-tuning the model parameters in order to improve performance. Ultimately, it will be important to carefully evaluate the strengths and limitations of different approaches in order to identify the most promising direction for future work.

### 4.3. Model reliability

Table 6 shows the agreement scores between the single-feature model and ensemble-feature models for different classifiers and a human expert. A comparison between the single-feature model and ensemble-feature models shows that the prediction of ensemble-feature models is more consistent than that of the single-feature model. For example, the agreement score of the model’s predictions by SVM single-feature models is 0.74, which is not so consistent with the human expert (0.96). The agreement score of ensemble models ranges from 0.79 to 0.82, the prediction by ensemble models is more similar to that of a human expert than that of a single-feature model, but still not consistent enough to replace a human expert.

**Table 6:** Cohen’s Kappa coefficient of different classifiers, compared between models using features from a single model, using our ensemble method and human expert.

Architecture	Model	Cohen’s Kappa coefficient
SVM	Single-Feature	0.74
SVM	Ensemble-Feature	**0.81**
LR	Single-Feature	0.73
LR	Ensemble-Feature	**0.79**
DT	Single-Feature	0.75
DT	Ensemble-Feature	**0.82**
Human expert		0.96

Here higher scores indicate better agreement among classifiers. Bold indicates better agreement.

We observed no significant difference in consistency among single-model classifiers using SVM, LR, and DT. The same is true when we used ensembles, but we found that the DT ensemble had slightly better performance than the SVM and LR ensembles, as reflected in agreement scores of 0.82 for LR, and 0.81 and 0.79 for SVM and LR, respectively.

The above assessment shows that ensemble models can be more accurate than a single model because they can capture a wider range of patterns and relationships in the data. In this case, it appears that the ensemble model has a higher agreement score than the single-feature model, which suggests that it is making more accurate predictions. This could also be due to the fact that the ensemble model is able to consider multiple features, rather than just one, which allows it to better capture the complexity of the data. Overall, the higher agreement score of the ensemble model indicates that it is a more effective model for the MI classification problem.

## 5. Conclusion

We proposed an accurate and robust deep learning-based approach for MI detection on echocardiograms. We showed that accurate segmentation models are not fully correlated with accurate MI classifiers, indicating that highly accurate segmentation of the LV is not a key factor for building an accurate MI detection system. We then proposed an ensemble method for combining multiple features provided by different LV segmentation models, which outperformed both single models and state-of-the-art methods on two echocardiogram datasets. Compared to these existing approaches, the proposed method demonstrated significant improvement across all evaluation metrics. We further illustrated that the proposed method shows a higher agreement score (Cohen’s kappa value) than single-feature methods, regardless of the features used. This high level of agreement suggests that our predictions are subject to less variation due to different feature extractors, making our method reliable and well-suited for use in the classification of MI.

Our work opens up several potential directions for further exploration. First, an end-to-end system for MI detection, rather than the current three-stage approach, should be developed. Second, further studies can be conducted to investigate the causes of performance drop on the external dataset and implement methods for addressing these issues. Third, it might be interesting to extend our training method into the pretraining stage to produce better pre-trained models. Additionally, further exploration of the factors that contribute to the success of ensemble learning in MI detection could be beneficial for improving the performance of future models.

## Data Availability

The raw data (the E-Hospital dataset) supporting the conclusions of this article will be made available by the authors, without undue reservation. The HMC-QU dataset used in this study can be found and freely downloaded on Kaggle at www.kaggle.com/datasets/aysendegerli/hmcqu-dataset.
